# Tin Foil

**Published:** 1881-11

**Authors:** 


*Tin Foil.*—The cervical wall of the proximal cavities in bicuspids and molars is *the* point about which we feel anxious. Many of our otherwise durable gold fillings fail there. As tin *seems* more preservative, of late we have been using in some cases a few folds of this metal to start the filling. Several years' test—the only true test is time—seems to encourage this procedure, so much so that it is occasionally applied to incisors where the cavities reach under the gums. If carefully used, it will not show. There is nothing new in this; indeed we believe the idea is so old as even to be new to the youngest; but it may save a tooth otherwise in doubt. Said a most excellent operator, lately: "twenty-five years experience has demonstrated that I can save more teeth with tin foil than with gold."

